# Vacuum-Deposited Donors for Low-Voltage-Loss Nonfullerene Organic Solar Cells

**DOI:** 10.1021/acsami.3c04282

**Published:** 2023-06-22

**Authors:** Pascal Kaienburg, Helen Bristow, Anna Jungbluth, Irfan Habib, Iain McCulloch, David Beljonne, Moritz Riede

**Affiliations:** † Clarendon Laboratory, Department of Physics, University of Oxford, Oxford OX1 3PU, U.K.; ‡ Department of Chemistry, Chemistry Research Laboratory, University of Oxford, Oxford OX1 3TA, U.K.; § KAUST Solar Center (KSC), King Abdullah University of Science and Technology (KAUST), Thuwal 23955, Saudi Arabia; ∥ Laboratory for Chemistry of Novel Materials, Center of Innovation and Research in Materials & Polymers (CIRMAP), University of Mons (UMONS), Mons B-7000, Belgium

**Keywords:** OPV, NFA, vacuum thermal evaporation, voltage loss, PHJ, bilayer

## Abstract

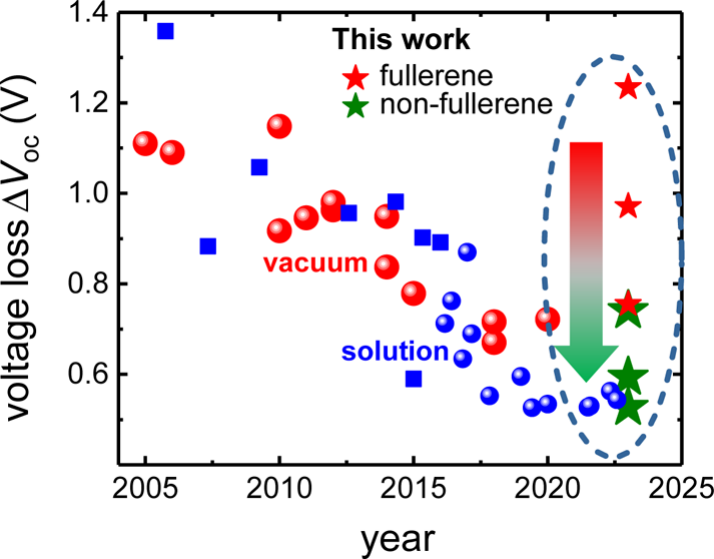

The advent of nonfullerene acceptors (NFAs) enabled records of organic photovoltaics (OPVs) exceeding 19% power conversion efficiency in the laboratory. However, high-efficiency NFAs have so far only been realized in solution-processed blends. Due to its proven track record in upscaled industrial production, vacuum thermal evaporation (VTE) is of prime interest for real-world OPV commercialization. Here, we combine the benchmark solution-processed NFA Y6 with three different evaporated donors in a bilayer (planar heterojunction) architecture. We find that voltage losses decrease by hundreds of millivolts when VTE donors are paired with the NFA instead of the fullerene C_60_, the current standard acceptor in VTE OPVs. By showing that evaporated small-molecule donors behave much like solution-processed donor polymers in terms of voltage loss when combined with NFAs, we highlight the immense potential for evaporable NFAs and the urgent need to direct synthesis efforts toward making smaller, evaporable compounds.

## Introduction

Organic photovoltaic (OPV) devices are typically prepared from solution or by thermal evaporation in vacuum.^1^ Solution-processed polymer/small-molecule (SM) blends produce certified record power conversion efficiencies (PCEs) in the laboratory exceeding 19%.^2−5^ However, transferring solution-processed laboratory records to upscaled industrial manufacturing faces several challenges^1,6−9^ such as halogen-free solvent processing in air, homogeneous and defect-free deposition across large areas, orthogonal solubility in multilayer and multijunction processing, device lifetime, batch-to-batch synthesis variation of polymeric components, and the high synthetic complexity of most high-performing molecules. Vacuum thermal evaporation (VTE) of OPVs relies on a mature deposition technology^1^—also applied in the very successful organic light-emitting diode industry—with straightforward additive manufacturing and multilayer stack design, homogeneous defect-free large-area deposition, and demonstration of long OPV lifetimes.^10^ Furthermore, by employing simpler molecular structures that can be easily synthesized to higher purity, VTE of OPV avoids or has overcome most of the barriers that solution fabrication faces in the context of industrial manufacturing. Indeed, Heliatek—employing VTE—has started producing on a 100–200 MWp/y production line, to our knowledge the largest running OPV production in the world. However, PCE laboratory records of VTE OPVs^11^ lack solution-processed ones.

**Figure 1:**
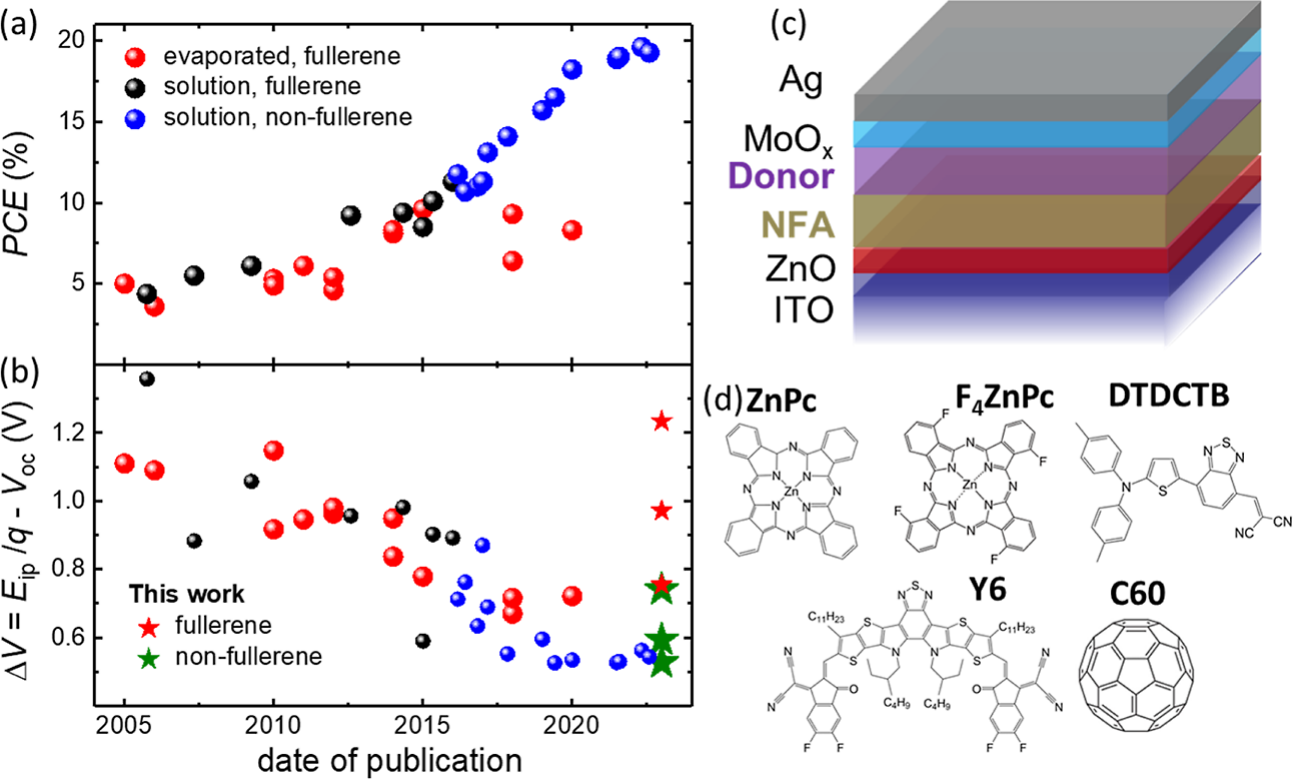
(a) Selected historic high-PCE reports from the literature comparing solution-processed fullerene- and NFA-based polymer blends with fullerene-based evaporated SM. The corresponding values are listed in Table S1. (b) Voltage losses of the same reports and bilayer data from this work. ZnPc devices show the highest and DTDCTB shows the lowest Δ*V*, see Table 1. (c) Bilayer planar heterojunction structure of solution-deposited Y6 NFA and the evaporated donor. (d) Molecules used in the study. Top: donors, bottom: acceptors, with C_60_ serving as a reference for the NFA.

The key driving factor for increased PCE of OPVs has regularly been the development of new molecules. Until about 2015,^9^ fullerene and its derivatives were the most efficient acceptor molecules and performance improvements were achieved by varying the donor molecule. Figure 1a shows that the PCEs of fullerene-based OPV saturated at about 11%^12^ for solution and around 10%^13,14^ for VTE OPVs. Since then, a broad class of nonfullerene acceptors (NFAs) have propelled PCEs of solution-processed polymer/SM systems close to 20%.^2−5^ The major trend behind improved PCEs is reduced open-circuit voltage loss as shown in Figure 1b. Here, we define voltage loss in a simple metric as the difference between the photovoltaic gap,^15,16^ i.e., the EQE inflection point (ip) at the long wavelength edge and open-circuit voltage Δ*V* = *E*_ip_ – *V*_oc_. For fullerene-based blends, solution and VTE-processed OPVs reduced voltage losses primarily by designing new donors. Subsequently, solution-processed polymer/SM reduced voltage losses further through NFA design. Most NFAs that are used in solution processing are too large to be evaporated, and to this day, no evaporable NFA-based VTE OPV has been reported that outperforms the equivalent device with fullerenes as acceptors^17−21^ (see Table S1). It is a priori unclear whether this is because designing such evaporable NFAs is challenging or because commonly used VTE donors are not compatible with evaporable NFAs with regard to high performance.

Here, we take a first step toward low-voltage loss NFA-based VTE OPVs by demonstrating that low-voltage losses can be achieved with common VTE donors. We do so by selecting the benchmark solution-processed NFA Y6^22−24^ and combining it with three different evaporated donors in a bilayer planar heterojunction architecture. We obtain significantly reduced voltage losses compared to equivalent fullerene reference devices. We achieve a lower voltage loss than any reported fully VTE device stack and match those of the best polymer/NFA systems while maintaining a reasonable *J*_sc_ and FF. By demonstrating that today’s VTE donors are suitable for low-voltage loss OPVs, we show that efforts need to focus on designing evaporable NFAs. Unlocking NFAs as a new class of highly efficient molecules for VTE as a readily scalable industrial OPV technology bears the tremendous potential to achieve real-world impact with OPVs in the context of sustainable development goals, particularly SDG7.

## Results

We fabricated bilayer stacks with a planar heterojunction of solution-processed acceptor/VTE donor consisting of ITO/ZnO (30 nm)/Y6 (30 nm)/F_*x*_ZnPc(20 nm)/MoO_*x*_(10 nm)/Ag(100 nm) as shown in Figure 1c. See the Experimental Methods section for the full chemical names of molecules. First, ZnO and Y6 were spin-coated from solution in air and N_2_, respectively, ensuring that we obtain comparable Y6 layers for all devices, followed by inert transfer into a vacuum deposition chamber where the donor and MoO_*x*_/Ag back hole contact was deposited. For the reference devices, Y6 was replaced with vacuum-deposited C_60_ of similar thickness as the typical acceptor in today’s VTE OPVs. We chose Y6 as an acceptor as it is considered a benchmark—and is arguably the most studied—NFA yielding good performance in solution-processed bulk heterojunctions (BHJs).^22−24^ Y6 shares the A–D–A structure as well as many benefits of a broader class of NFAs. Using ZnPc and its fluorinated version F_4_ZnPc as evaporated donors, shown in Figure 1d, allowed us to study the influence of different interface energetics, with F_4_ZnPc energy levels shifted away from vacuum compared to ZnPc^25^ while leaving other properties such as absorption onset and extinction coefficient unchanged. We focus our discussion mostly on ZnPc and F_4_ZnPc, showing the role that energy levels play, while DTDCTB data is shown mostly in the Supporting Information.

**Figure 2:**
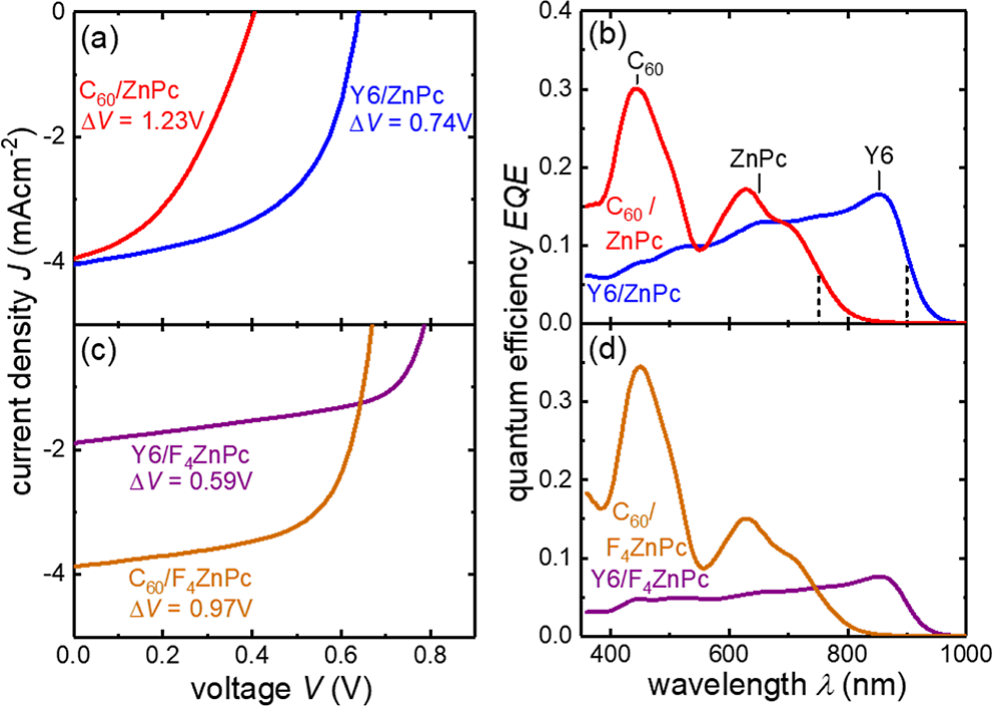
Solar cell (a,c) current density–voltage characteristics and (b,d) EQE of bilayer planar heterojunctions with (a,b) ZnPc and F_4_ZnPc (c,d). The current densities are mismatch-corrected to AM1.5G equivalent intensity. The dashed lines in (b) indicate the inflection point taken as a reference for the band gap. Peak labels indicate the molecules’ contribution to absorption. The assignment is supported by spectroscopic ellipsometry measurements in Figure S1.

Strikingly, the Y6/ZnPc solar cell shown in (Figure 2a) and listed in Table 1 shows a significantly higher *V*_oc_ than the C_60_/ZnPc reference device despite having a lower photovoltaic gap. Taking the gap as the inflection point of the EQE spectra^15,16^ shown in Figure 2b, the voltage loss of the Y6/ZnPc device, Δ*V* = 740 mV, is 500 mV lower than the C_60_/ZnPc reference. While certain fullerene-based VTE OPVs in Figure 1b achieve Δ*V* < 740 mV, this shows that evaporable NFAs, once identified, will likely enable lower voltage losses and higher device performances for a wider range of donor molecules since the major restriction of specifically matching C_60_’s energy levels is lifted. To further reduce voltage losses in the bilayer device, we employ F_4_ZnPc as the donor in Figure 2c,d, whose energy levels are shifted further away from the vacuum level by about 700 meV compared to ZnPc^25^ (see Figure S6). The C_60_/F_4_ZnPc reference shows an improved open-circuit voltage *V*_oc_ = 0.67 V compared to the C_60_/ZnPc device with *V*_oc_ = 0.41 V. The Y6/F_4_ZnPc device achieves a higher *V*_oc_ = 0.79 V than the fullerene reference, and its voltage loss of Δ*V* = 590 mV is even lower than that of Y6/ZnPc. This value is lower than any of the high-performing VTE devices shown in Figure 1b, where this study’s new data points are contained. Finally, in analogy to a recent literature report,^26^ we fabricated bilayers with DTDCTB as a donor, shown in Figure S2, which, with Y6, reduces voltage losses further to Δ*V* = 530 mV.

**Table 1:** Device Performance^a^

	*E*_g_ (eV)	*V*_oc_ (V)	Δ*V* (V)	FF (%)	*J*_sc_^AM1.5^ (mA/cm^2^)	PCE^AM1.5^ (%)	AM1.5G equiv intensity
C_60_/ZnPc	1.64	0.41	1.23	41	3.9	0.7	0.96
Y6/ZnPc	1.38	0.64	0.74	55	4.0	1.4	1.38
C_60_/F_4_ZnPc	1.64	0.67	0.97	63	3.9	1.6	0.94
Y6/F_4_ZnPc	1.38	0.79	0.59	54	1.9	0.8	1.36
C_60_/DTDCTB	1.57	0.81	0.76	53	4.0	1.7	0.94
Y6/DTDCTB	1.38	0.85	0.53	35	3.3	1.0	1.38

a PCE and *J*_sc_ are mismatch-corrected to AM1.5G equivalent intensity. The AM1.5G equivalent intensity (suns equivalent) during measurement is given. The EQE inflection point is taken for the photovoltaic band gap *E*_g_.^15,16^

Before discussing voltage losses in more detail, we turn to the photocurrent density of the devices. Bilayers are expected to yield lower short-circuit current densities than BHJs because only photons absorbed within roughly one exciton diffusion length of the planar donor/acceptor interface will contribute efficiently to free charge carrier generation. Additionally, for a combined bilayer thickness of only 50 nm and in the absence of an optical spacer, most absorption will occur away from the interface. Recent reports have observed some degree of free charge generation in Y6-only films with spectroscopic methods.^27,28^ The reported device performance of around PCE = 0.5% is significantly lower than what we report here (1.4% for Y6/ZnPc). Since different Y6/donor pairing leads to vastly different photocurrents and voltage losses, despite identical Y6 layers—they are all processed on the same surface with the same parameters so that the morphology of the Y6 is comparable across all samples—self dissociation in Y6 is very unlikely to be the dominant mechanism of photovoltaic action. To further demonstrate that the main contribution to the observed photocurrent indeed stems from the interface, we fabricated Y6 layers of the same thickness without any donor evaporated on top. The performance (PCE < 0.1%) and *J*_sc_ (0.3 mA/cm^2^) shown in Figure S3 are much lower than the *J*_sc_ of the NFA bilayer devices we report here (between 2 and 4 mA/cm^2^). We note that the bilayer device performance not only depends on the donor–acceptor pairing but will be influenced by the microstructure of the bilayer interface, namely, the roughness of the interface,^29^ the degree of order, and the molecular orientation,^30^ which can be tuned during fabrication. Indeed, the electronic properties of Y6 have been shown to depend on processing conditions^30−32^ and careful device optimization may yield better exciton diffusion length in Y6 and an improved interface morphology. A fully solution-processed bilayer of Y6/CuSCN, where an interface with CuSCN facilitates the splitting of excitons, achieved similar performance.^33^ With the same material pairing, 4.5% PCE was achieved after optimizing processing conditions.^34^ To ensure comparability within our study, all Y6 layers in this work were coated in the same way. Li and Lin^26^ discussed the benefits of using a bilayer planar heterojunction architecture over conventional BHJs by fabricating devices similar to ours, employing Y6 and different evaporated donors, and found reduced voltage losses for the planar vs BHJs. In support of our findings, the overall performance in terms of short-circuit current density *J*_sc_ and voltage loss (down to 550 mV) matches ours.

The *J*_sc_ of our C_60_/F_4_ZnPc and C_60_/ZnPc devices is 3.9 mA/cm^2^ in both cases as expected from essentially identical absorption spectra and previous literature.^35,36^ However, Y6/F_4_ZnPc shows a decreased *J*_sc_ of 1.9 mA/cm^2^ compared to the Y6/ZnPc (4 mA/cm^2^) despite no change in absorption spectra indicated by the similar shape of the EQE spectra. This indicates a drop in exciton splitting efficiency for the fluorinated donor, which is well known for systems where energetic offsets are insufficient.^37^ See Figure S6 for a qualitative discussion of molecular energy levels. Figure S3 shows a Y6/ZnPc/F_4_ZnPc device where ZnPc and F_4_ZnPc are blended in a 1:1 ratio, leading to energy levels of the “alloyed” blend in between the individual components.^25^ The resulting *J*_sc_ and *V*_oc_ of 0.69 V is in between the values obtained for the individual components, confirming the trend of *V*_oc_ and *J*_sc_ with donor energy levels—namely, that lower offsets result in a lower voltage loss, but exciton splitting becomes less efficient and the photocurrent decreases. We note that excitons still get separated at the Y6/F_4_ZnPc interface, leading to an appreciable *J*_sc_ but that a detailed analysis of the exciton separation efficiency is beyond the scope of this study. The trade-off between *V*_oc_ and photocurrent for low energetic offsets observed in our devices serves as further evidence that the donor/acceptor interface is the dominant origin for the attained values of *V*_oc_ and photocurrent (as opposed to photovoltage and photocurrent originating in the bulk of Y6). Importantly, the high FF > 50% of the Y6/ZnPc bilayer—larger than the corresponding C_60_ device with 41%—suggests that exciton dissociation is not heavily reliant on the electric field, which was the case for previously reported^20^ fullerene-free evaporated OPVs as shown in Table S2. The Y6/F_4_ZnPc and Y6/DTDCTB devices, showing lower voltage loss than Y6/ZnPc, on the other hand, have lower FFs than the corresponding devices with C_60_. Hence, the investigated Y6/VTE donor combinations demonstrate that voltage loss can be reduced, compared to corresponding C_60_ devices, without compromising the *J*_sc_ and FF significantly. For further reduced voltage loss, there is a trade-off between reduced losses in *V*_oc_ and increased losses in the *J*_sc_ and FF due to less efficient, voltage-dependent free charge generation.

**Figure 3:**
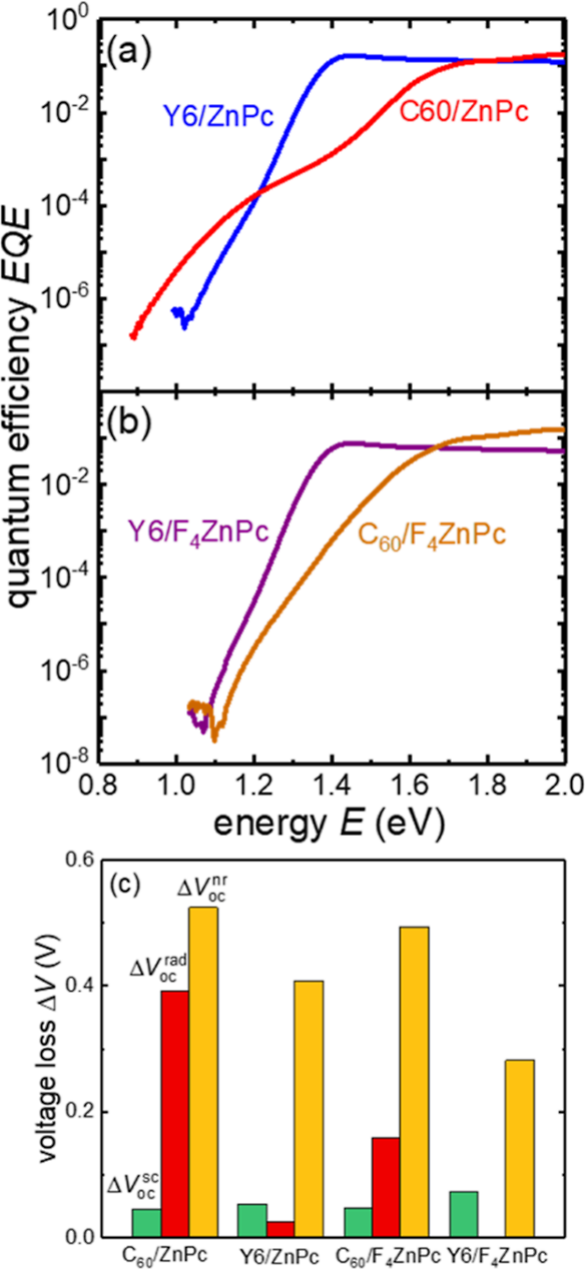
Sensitive EQE of (a) ZnPc and (b) F_4_ZnPc-based bilayer devices. (c) Resulting open-circuit voltage losses split into losses due to reduced photocurrent Δ*V*_oc_^sc^, radiative Δ*V*_oc_^rad^, and nonradiative Δ*V*_oc_^nr^ losses. The corresponding values are listed in Table S4.

Next, in Figure 3, we investigate voltage losses and charge transfer (CT) states via sensitive external quantum efficiency (sEQE) measurements over a wide dynamic range of about 7 orders of magnitude. The Y6/ZnPc and Y6/F_4_ZnPc spectra in Figure 3a,b, respectively, show a steep tail without any discernible shoulder. On the other hand, C_60_/ZnPc shows a clear CT state shoulder and F_4_ZnPc/C_60_ shows an overall rounded absorption edge and gradual onset. The sEQE of the fullerene-based bilayers qualitatively matches that of the corresponding BHJs,^36,38^ while the Y6-based bilayers are qualitatively similar to PM6/Y6 blends,^39,40^ pointing to similar behavior of SM donors and polymers when combined with Y6 as a benchmark NFA. Gaussian fits of the sEQE spectra according to Marcus theory, shown in Figure S4 and listed in Table S3, quantify the reduced energetic offset between the lowest singlet S1 and CT state for Y6 compared to C_60_ devices. While the fits reproduce the experimental data well, some uncertainty cannot be avoided when fitting sEQE tails without clear features.^41^

For a reliable quantitative analysis, we dissect voltage losses in terms of radiative and nonradiative voltage losses in Figure 3c and Table S4 following Rau’s detailed balanced analysis.^15,41,42^ Here, radiative losses are understood as losses in *V*_oc_^rad^ relative to the radiative limit *V*_oc_^SQ^, hence reflecting subgap absorption, e.g., by the CT state. See the Experimental Methods section for details. Note that since photocurrent generation in our bilayer devices is limited, *V*_oc_ losses of tens of millivolts resulting from *J*_sc_ losses^15,43^ (Δ*V*_oc_^sc^) are considerable. C_60_/ZnPc shows large nonradiative and radiative voltage losses of 520 and 390 mV, respectively, with the latter being reduced to 160 mV for C_60_/F_4_ZnPc, matching our reported behavior of corresponding BHJs.^36^ The radiative voltage loss drops significantly for Y6/ZnPc to 30 mV, which is lower than any of the VTE systems we have investigated previously.^36^

The nonradiative loss in Y6/F_4_ZnPc of 280 mV is ∼200 mV smaller than that in C_60_/F_4_ZnPc, even though *V*_oc,rad_ is 100 mV lower in Y6/F_4_ZnPc. This trend contradicts the energy gap law, which predicts higher nonradiative loss for lower *V*_oc,rad_.^44,45^ In a further deviation from the energy gap law, Y6/ZnPc shows >100 mV higher nonradiative voltage loss than Y6/F_4_ZnPc, despite having almost identical *V*_oc,rad_. A detailed investigation of the origins of this behavior and calculations of the molecular energy levels is beyond the scope of this work. We note that we previously demonstrated high nonradiative losses of F_*x*_ZnPc:C_60_ blends compared to other SM VTE donors^36^ and that even lower nonradiative losses have been demonstrated for similar bilayer devices with DTDCTB as an evaporated donor^26^ (see also Figure S2). To conclude, reduced nonradiative voltage losses that do not follow the energy gap law, likely due to CT-S1 hybridization,^39,46^ are a further beneficial feature of polymer/NFA blends^9^ that may translate to NFA/VTE-SM systems.

Both Y6/ZnPc and Y6/F_4_ZnPc can be classified as low-loss systems with little radiative losses, which is unprecedented for VTE OPVs. Both the vanishing radiative loss and the reduced nonradiative loss of Y6/F_4_ZnPc match losses observed in PM6/Y6 blends.^40^ Altogether, our findings suggest that in terms of voltage loss, VTE-SM donors behave very much like donor polymers when paired with NFAs, so similar physical models are applicable and similar high performance may be expected in the future.

## Conclusions

We paired commonly used vacuum thermally evaporated donor molecules, ZnPc, F_4_ZnPc, and DTDCTB, with the solution-processed benchmark NFA Y6 in a bilayer (planar heterojunction) device architecture. We found that the SM donors behave much like donor polymers when paired with Y6 in terms of voltage loss. Most significantly, we find low radiative voltage losses of few tens of millivolts which have not been achieved with fullerene-based VTE OPVs. At the same time, the low-voltage loss does not seem to compromise the *J*_sc_ and FF. We also find reduced nonradiative losses of NFA/VTE-SM compared to those of C_60_/VTE-SM systems and indications that the investigated systems outperform predictions from the energy gap law, likely due to hybridization of singlet and CT states. Our solution-NFA/VTE-SM donor devices yield lower overall voltage loss than any reported fullerene VTE system and match those of high-performing polymer/NFA blends. Our work demonstrates that commercially available SM donors are suitable to be paired with NFAs to benefit from the various advantages of NFAs demonstrated in solution-processed OPVs. Expected benefits of evaporable NFAs compared to fullerenes include low-voltage losses, broadened absorption range, an increased parameter space of molecular properties, as well as a higher degree of freedom in the design of donor molecules since their energy levels are not restricted by having to match those of C_60_.

Our findings highlight the tremendous potential and the urgent need to synthesize evaporable NFAs. Developing design strategies for evaporable NFAs can be assisted by recently gathered insights into the working mechanisms of solution-processed NFA blends.^9,47^ Packing dimensionality, long exciton lifetimes, quadrupolar moments, the small band gap of the NFA, and donor–acceptor Förster energy transfer may play an important role in high-efficiency NFA blends. Finally, the question needs to be answered whether the extended size of currently available NFAs, making them too large for sublimation in vacuum, is an essential feature for enabling their high performance or a coincidental design result. In the quest for efficient evaporable NFAs, it might be worth studying earlier generations of solution-processed NFAs, as well as efforts to synthesize smaller solution-based NFAs with lower synthetic complexity.

## Experimental Methods

### Materials and Substrates

Full chemical names of molecules: Y6: 2,2′-((2*Z*,2′*Z*)-((12,13-bis(2-ethylhexyl)-3,9-diundecyl-12,13-dihydro-[1,2,5]thiadiazolo[3,4-*e*]thieno[2″,3″:4′,5′]thieno[2′,3′:4,5]pyrrolo[3,2-*g*]thieno[2′,3′:4,5]thieno[3,2-*b*]indole-2,10-diyl)bis(methanylylidene))bis(5,6-difluoro-3-oxo-2,3-dihydro-1*H*-indene-2,1-diylidene))dimalononitrile; ZnPc: zinc phthalocyanine; F_4_ZnPc: zinc(II)-1,8,15,22-tetrafluoro-29*H*,31*H*-phthalocyanine; DTDCTB:2-((7-(5-(di-*p*-tolylamino)thiophen-2-yl)benzo[*c*][1,2,5]thiadiazol-4-yl)methylene)malononitrile; BPhen: 4,7-diphenyl-1,10-phenanthroline; ITO (20 Ω/sq on Eagle XG glass, rms roughness < 7 Å) was purchased from Thin Film Devices TFD Inc., USA. Fullerene-C_60_ was purchased from Creaphys GmbH, Germany, in its optoelectronic grade (sublimed multiple times). ZnPc, F_4_ZnPc, DTDCTB, and BPhen were purchased in a sublimed grade from Luminescence Technology Corp., as well as MoO_3_. All reagents for the zinc oxide precursor were purchased from Sigma-Aldrich. Y6 was obtained from Brilliant Materials.

### Sample Fabrication

The layer stack is the ITO/ZnO/acceptor (Y6 or C60)/donor (ZnPc, F_4_ZnPc, or DTDCTB)/BPhen/Ag. All substrates were cleaned for 10 min in an ultrasonic bath of a 2.5% Hellmanex solution, followed by DI water, acetone, and isopropanol. Prior to zinc oxide deposition, the substrates were UV ozone-treated for 7 min. Zinc oxide: A solution of zinc acetate dihydrate (500 mg) with ethanolamine (140 μL) in 2-methoxy ethanol (5 mL) was made up the night before coating. On the day of coating the solution was filtered using a PTFE filter with a 0.45 μm pore size before spin coating at 6000 rpm/45 s in air. The films were thermally annealed at 200 °C/20 min. After the substrates had cooled to room temperature, a cosolvent of ethanolamine (250 μL) and 2-methoxy ethanol (140 μL) was spin-coated at 6000 rpm/45 s and annealed at 140 °C/10 min. The Y6/Y6 solution (6 mg/mL in chloroform) was dissolved at room temperature overnight. The Y6 solution was dynamically spin-coated at 1500 rpm/30 s in a N_2_-filled glovebox. After Y6 deposition, the samples were transferred into a vacuum chamber (EVAP300, Creaphys, base pressure 10^–7^ mbar), where all subsequent layers were thermally evaporated followed by transfer into an N_2_-filled glovebox without vacuum break for encapsulation. Layers with nominal thickness, determined from tooled quartz crystal microbalances when evaporated, were ZnO (∼30 nm), Y6 (∼30 nm), C_60_ (30 nm, 0.3 Å/s), donor (20 nm, 0.3 Å/s), BPhen (8 nm, 0.1 Å/s), and Ag(∼100 nm, 1 Å/s). The solar cells had an active area of 0.08 cm^2^ defined by the geometric overlap between ITO and Al, and there were eight solar cells per substrate.

### *J*–*V*

Current density–voltage characteristics were measured under illumination from a Newport Oriel Sol3A solar simulator with a Xe arc lamp. The listed values for *J*_sc_ and PCE are after spectral mismatch correction^48^ carried out postmeasurement and included an intensity correction to a 100 mW/cm^2^ equivalent. Best-performing samples out of ∼18 are chosen for this report.

### External Quantum Efficiency

Sensitive EQE measurements were performed using a custom-built setup. White light from a tungsten-halogen light source (Princeton Instruments, TS-428, 250 W) was diffracted by wavelength using a monochromator (Princeton Instruments, Spectra-Pro HRS300, Triple Grating Imaging Spectrograph). Using spectral filters (Thorlabs, edge pass and long pass filters), stray light and higher-order diffractions were removed. The light was modulated using a chopper wheel (Stanford Research Systems, SR450, Optical Chopper) before being focused onto the device under testing. The resulting photocurrent was preamplified (Zürich Instruments, HF2TA Current Amplifier) before being read out by a Lock-In amplifier (Zürich Instruments, HF2LI Lock-In Amplifier).

### Spectroscopic Ellipsometry

Spectroscopic ellipsometry was carried out with a Woollam RC2 spectroscopic ellipsometer at 55, 65, and 75° angles of incidence. Single-component films on glass were prepared in a similar fashion to the devices. The acquired ψ and Δ spectra were model-fitted with the CompleteEASE software from J.A. Woollam company to obtain the optical constants *n* and κ. Using B spline models, anisotropic fits were performed, yielding in-plane and out-of-plane components and the results were confirmed by matching transmission data.

### Voltage Loss Calculation

We follow the detailed balance analysis by Rau et al.^15,41^ The optical band gap *E*_g_ was defined as the inflection point of the EQE absorption edge^15^ on a linear scale. Detailed balance theory^49^ yields *V*_oc_^SQ^ and *J*_sc_^SQ^. The losses from imperfect photocurrent were calculated as Δ*V*_oc_^sc^ = *k*_B_*T*/*q*·ln(*J*_sc_^SQ^/*J*_sc_). The radiative *V*_oc_^rad^ was calculated assuming reciprocity between EQE and electroluminescence^42,50^ via . Here, ϕ_bb_ is the black-body spectrum and *E*_min_ was chosen small enough such that *V*_oc_^rad^(*E*_min_) saturates as discussed in Figure S5. The remaining loss terms were then calculated via Δ*V*_oc_^SQ^ = *E*_g_ – *V*_oc_^SQ^, Δ*V*_oc_^rad^ = *V*_oc_^SQ^ – Δ*V*_oc_^sc^ – *V*_oc_^rad^, and Δ*V*_oc_^nr^ = *V*_oc_^rad^ – *V*_oc_. Finally, the LED quantum efficiency is obtained from Δ*V*_oc_^nr^ = *k*_B_*T*/*q*·ln(*Q*_LED_^–1^).

### CT State Fitting

EQE spectra were fit with Marcus theory , with CT state energy *E*_CT_, reorganization energy λ, and oscillator strength *f*_osc_. The singlet state S1 was fitted first, and then the CT state was fitted to the low-energy region of the difference between the data and the S1 fit.^20,41,51^

## Supporting Information Available

Values cited and depicted in the article, in-plane extinction coefficients, additional device *J*–*V* and (s)EQE characteristics, CT state fits, details on the voltage loss calculations, and discussion of molecular energy levels (PDF)
