# Supplementary material for: Vacuum-Deposited Donors for Low-Voltage-Loss Nonfullerene Organic Solar Cells
Source: ACS Appl Mater Interfaces. 2023 Jun 22;15(26):31684–91. doi: 10.1021/acsami.3c04282 (PMC10326856; doi:10.1021/acsami.3c04282)
Supplement: Supplementary file 1 — am3c04282_si_001.pdf [file am3c04282_si_001.pdf]

Supporting information

for

Vacuum-deposited donors for low-voltage loss non-fullerene organic solar cells

Authors:

Pascal Kaienburg[1]*, Helen Bristow[2], Anna Jungbluth[1], Irfan Habib[1], Iain McCulloch[2,3], David Beljonne[4], Moritz Riede[1]

Affiliations

[1] Clarendon Laboratory, Department of Physics, University of Oxford, Oxford OX1 3PU, UK

[2] Department of Chemistry, Chemistry Research Laboratory, University of Oxford, Oxford OX1 3TA, UK

[3] King Abdullah University of Science and Technology (KAUST), KAUST Solar Center (KSC), Thuwal 23955, Saudi Arabia

[4] Laboratory for Chemistry of Novel Materials, Center of Innovation and Research in Materials & Polymers (CIRMAP), University of Mons (UMONS), B-7000 Mons, Belgium

* Corresponding author: pascal.kaienburg@physics.ox.ac.uk

Table S1: Performance values of data presented in fig.1. The photovoltaic gap is estimated in the same way as the experimental data in this work via the inflection point E_{ip} of the EQE.

year	blend	d [nm]	E_{ip} [eV]	V_{oc} [V]	J_{sc} [mA/cm^2]	FF [%]	PCE [%]	ΔV [V]	ref.
**solution, non-fullerene**									
2001	MDMO-PPV:PC60BM	100	2.28	0.820	5.3	61	2.5	1.46	1
2006	P3HT:PC60BM	220	1.97	0.610	10.6	67	4.4	1.36	2
2007	PCPDTBT:PC70BM	110	1.50	0.620	16.2	55	5.5	0.88	3
2009	PCDTBT:PC70BM	80	1.94	0.880	10.6	66	6.1	1.06	4
2013	PTB7:PC70BM	80	1.71	0.754	17.5	70	9.2	0.96	5
2014	PPDT2FBT:PC70BM	290	1.77	0.790	16.3	73	9.4	0.98	6
2015	PNOz4T:PC71BM	225	1.55	0.960	14.5	64	8.5	0.59	7
2015	PNTz4T:PC71BM	290	1.61	0.708	19.4	73	10.1	0.90	8
2016	PffBT4T:PC71BM	350	1.68	0.784	19.8	73	11.3	0.89	9
**solution, non-fullerene**									
2016	PBDB-T:ITIC	100	1.66	0.902	16.7	71	10.7	0.76	10
2016	PBDB-T:IT-M:Bis[70]PCBM	100	1.66	0.952	17.4	74	11.8	0.71	11
2017	PCE10:IDTBR:IDFBR	90	1.66	1.030	17.2	60	11.0	0.63	12
2017	PTB7-Th:BTR:PC71BM	250	1.62	0.751	21.4	70	11.3	0.87	13
2017	PBDB-T-SF:IT-4F	100	1.57	0.880	20.9	71	13.1	0.69	14
2018	PTB7-Th:COi8DFIC:PC71BM	108	1.25	0.700	28.8	71	14.1	0.55	15
2019	PM6:Y6	150	1.43	0.830	25.3	75	15.7	0.60	16
2019	PBDB-TF:BTP-4Cl	100	1.39	0.867	25.4	75	16.5	0.53	17
2020	D18:Y6	103	1.39	0.859	27.7	77	18.2	0.53	18
2022	PBDB-TF:HDO-4Cl:eC9	100	1.39	0.866	27.1	81	18.9	0.53	19
2022	PBQx-TF:eC9-2Cl:F-BTA3	100	1.41	0.879	26.7	81	19.0	0.53	20
2022	PM6:D18:L8-BO	120	1.46	0.896	26.7	82	19.6	0.56	21
2023	PM6:L8-BO:BTP-S10	100	1.44	0.898	26.8	80	19.3	0.54	22
**evaporated, fullerene**									
2005	CuPc:C60	10	1.65	0.540	15.0	61	5.0	1.11	23
2006	ZnPc:C60	15	1.65	0.560	8.4	61	3.6	1.09	24
2010	H2PC:C60	960	1.55	0.402	18.3	53	5.3	1.15	25
2010	DCV6T:C60	40	1.80	0.880	11.6	57	4.9	0.92	26
2011	HB194:C60	55	1.91	0.962	12.6	47	6.1	0.95	27
2012	F4ZnPC:C60	65	1.65	0.690	12.1	55	4.6	0.96	28
2012	SubPC:C70	54	2.00	1.020	12.9	41	5.4	0.98	29
2014	DBP:C70	54	1.88	0.930	13.2	66	8.1	0.95	30
2014	DCV5T-Me:C60	40	1.80	0.960	13.2	66	8.3	0.84	31
2015	DTDCPB:C70	80	1.70	0.920	15.8	67	9.6	0.78	32
2018	iBuBTDC:C70	80	1.61	0.940	16.5	60	9.3	0.67	33
2018	BDP-2:C60	50	1.48	0.760	13.6	62	6.4	0.72	34
2020	DCPNT:C70	60	1.63	0.910	14.3	60	8.3	0.72	35

Table S2: Performance of evaporated BHJ and PHJ that do not use fullerene as acceptor. The 2019 report by Ullbrich et al.[36] used wide-gap molecules resulting in a high lying CT state, resulting in low

non-radiative losses in agreement with the energy gap law. However, overall voltage loss is still significant due to the S_1-CT offset. The efficiency of a newly synthesized evaporable NFA in 2021 by Yue et al.[37] lags in efficiency and shows a voltage loss comparable to fullerene. By exploiting unique properties of the SubNc class of molecules[38,39] , the 2014 report by Cnops et al.[40], also known as 'the IMEC stack', achieved the highest performance for PHJ VTE OPV but voltage loss does not go below the best reported fullerene-based BHJs. With the same class of molecules, a remarkably low voltage loss, comparable to the values attained in this work, was achieved by Nikolis et al.[38] in 2020, while maintaining high J_{sc}. However, the *FF* of only 33% could only be recovered by replacing SubPc with Cl6-SubPc, which increased voltage losses. These reports highlight that there is not only a trade-off of low voltage loss with high J_{sc}, but also with high *FF*. The likely reason is voltage-dependent exciton dissociation, i.e. free charges might be generated efficiently at J_{sc}, but not at the maximum power point anymore. Efficient solution-processed NFA-based blends in Table S.I do not seem to be limited by this trade-off between low voltage loss and high *FF*. Importantly, the high *FF*>50% for the bilayers reported in this work also seems to avoid the trade-off.

year	blend	d [nm]	E_g [eV]	V_{oc} [V]	J_{sc} [mA/cm^2]	*FF* [%]	*PCE* [%]	ΔV [V]	ref.
**evaporated, non-fullerene**									
2019	BF-DPB:B4PYMPM	30	3.02	2.04	0.6	70	0.86	0.98	36
	BF-DPB:TmPPPyTz	30	3.02	2.13	0.2	20	0.09	0.89	36
2021	DTDCTB/CBD	20/40	1.48	0.77	2.6	34	0.67	0.71	37
2014	a-6T/SubNc/SubPc	60/12/18	1.70	0.96	14.6	61	8.4	0.74	40
2020	SubNc/SubPc	12/18	1.70	1.14	7.3	33	2.8	0.56	38
	SubNc/Cl6-SubPc	12/18	1.70	1.03	7.4	60	4.5	0.67	38

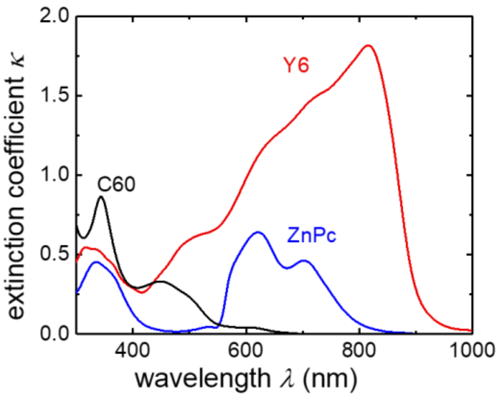

Figure S1: In-plane extinction coefficient measured via spectroscopic ellipsometry to support the interpretation of *EQE* data. See experimental method section for details of the analysis.

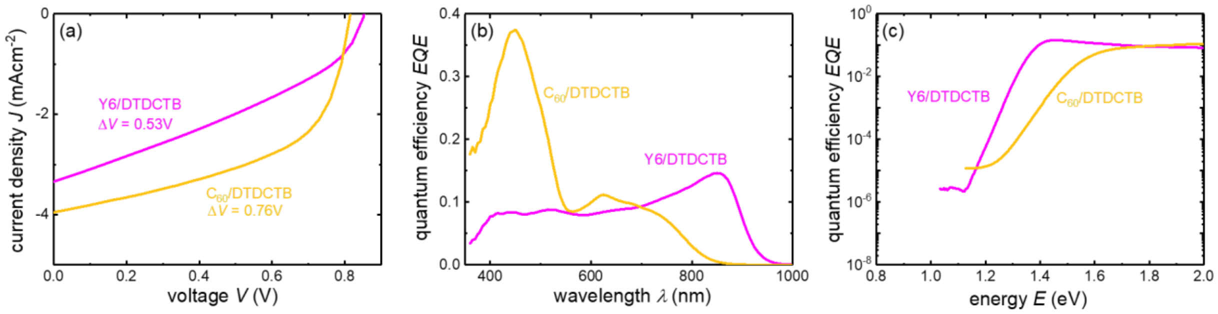

Figure S2: Device characteristics for DTDCTB as donor. (a) to AM1.5g equivalent intensity mismatch corrected *J*-*V* and *EQE* on a (b) linear, and (c) logarithmic scale.

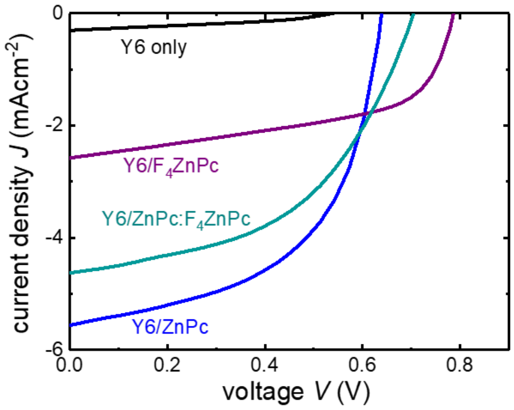

Figure S3: Additional *J*-*V* characteristics. The data here is not mismatch corrected but comparable across samples due to similar absorption spectra. The performance parameters for the Y6 only device without donor are $V_{\text{oc}} = 0.54\text{V}, J_{\text{sc}} = 0.30\text{mA/cm}^2,\ FF = 35\%,\ PCE = 0.06\%$. The Y6/ZnPc and Y6/$F_4$ZnPc is the same, but uncorrected, data as shown in the main text. The J_{sc} and V_{oc} of the Y6/ZnPc:F_4ZnPc (1:1) device lies in between those of the unblended F_xZnPc devices, demonstrating the importance of energy level offset at the bilayer interface for V_{oc} and photocurrent generation.

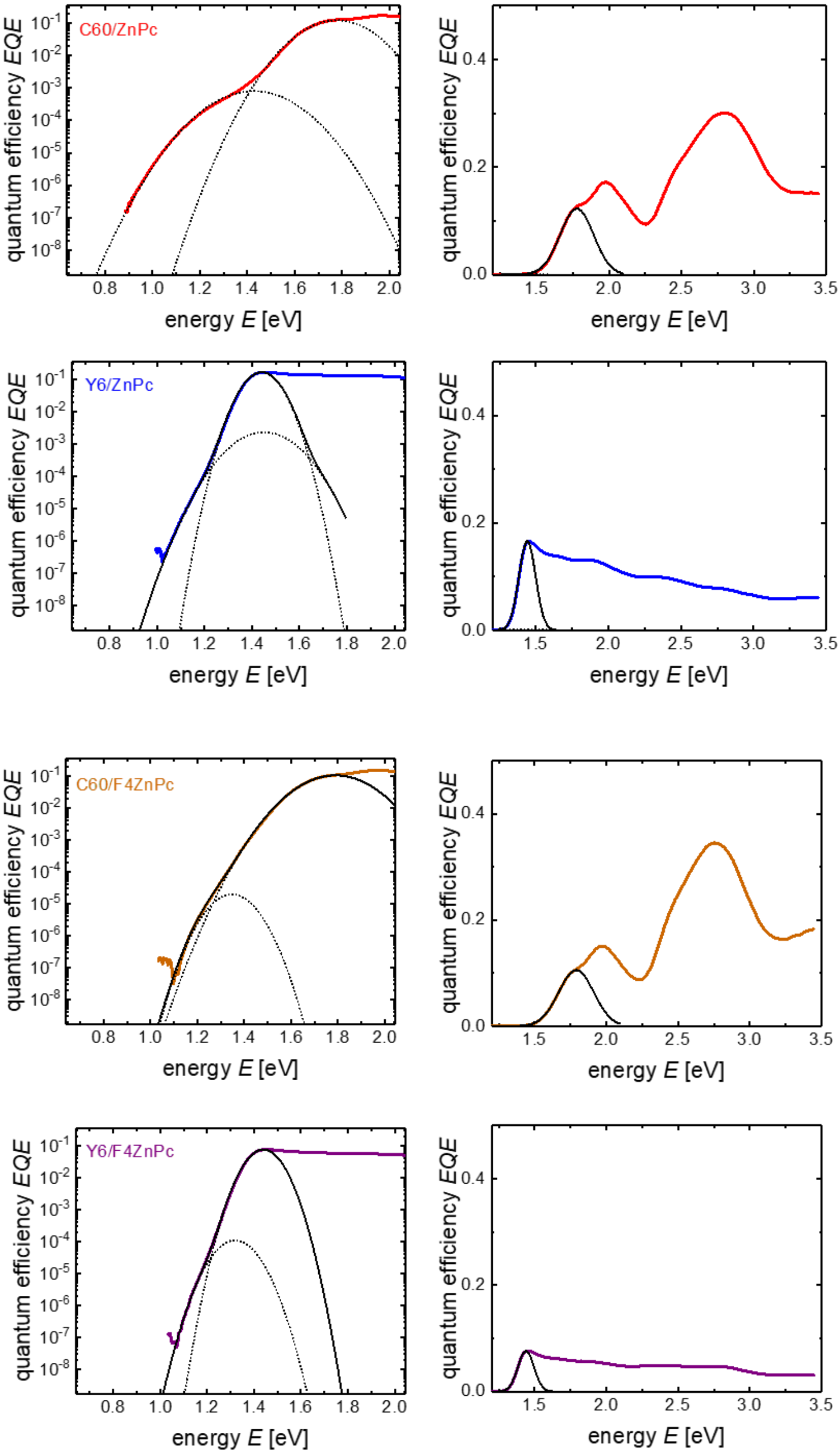

Figure S4: Fits of sEQE data with Gaussian singlet and CT states as described in the Experimental Methods section. The values of the fit results are given in Table SII.

Table S3: Results of CT fits in fig. S4 in a Marcus model with CT state energy E_{CT}, reorganization energy λ, and oscillator strength f_{osc} as described in the Experimental Methods section.

	E_{CT} (eV)	λ_{CT} (eV)	$f_{\mathrm{osc}}^{\mathrm{CT}}$ (eV^2)	E_{S1} (eV)	λ_{S1} (eV)	$f_{\mathrm{osc}}^{\mathrm{S1}}$ (eV^2)	ΔE_{CT} (eV)
C_{60}/ZnPc	1.11	0.33	4E-04	1.52	0.26	6E-02	0.41
Y6/ZnPc	1.27	0.19	8E-04	1.38	0.06	3E-02	0.11
C_{60}/F_4ZnPc	1.25	0.10	5E-06	1.51	0.29	6E-02	0.26
Y6/F_4ZnPc	1.24	0.08	2E-05	1.38	0.06	2E-02	0.14

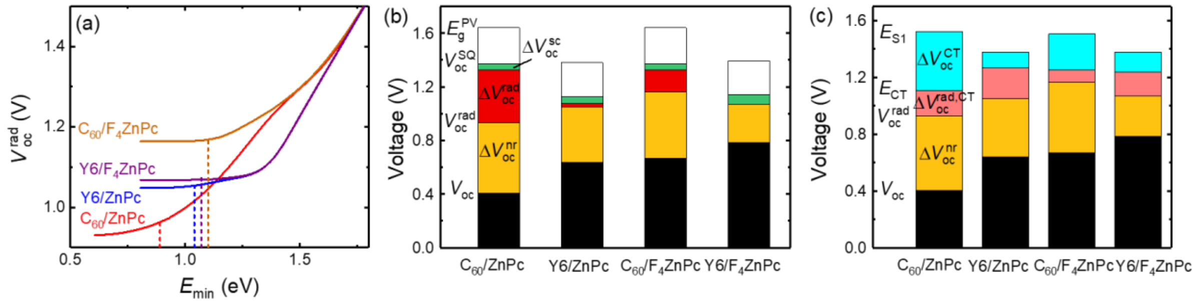

Figure S5 (a) Contribution to $V_{\mathrm{oc}}^{\mathrm{rad}}$ of different parts of the *EQE* spectrum according to the equation given in the Experimental Methods section. The vertical lines indicate the energies where the experimental EQE data hit the noise floor and deviate from the CT fits. For energies below this point, the fits were used to calculate further contributions to $V_{\mathrm{oc}}^{\mathrm{rad}}$. The $V_{\mathrm{oc}}^{\mathrm{rad}}$ value is almost saturated and extending the measured curve by well-fitted data only corrects its value by ~10meV. (b) Same data as in fig. 3(c) as stacked bar diagram. (c) Voltage losses in the energetic state picture[41,42] highlighting the importance of the CT state for the V_{oc} in OPV. Here, $q\Delta V_{\mathrm{oc}}^{\mathrm{CT}} = E_{\mathrm{S1}} - E_{\mathrm{CT}}$, where E_{S1} and E_{CT} are the energies of the lowest lying singlet and the CT state, respectively. The radiative loss $\Delta V_{\mathrm{oc}}^{\mathrm{rad,CT}} = q^{-1}E_{\mathrm{CT}} - V_{\mathrm{oc}}^{\mathrm{rad}}$ is defined differently from the detailed balance picture which is the focus in the main text. $V_{\mathrm{oc}}^{\mathrm{rad}}$ and $\Delta V_{\mathrm{oc}}^{\mathrm{nr}}$ are defined in the same way for both analyses.

Table S4: Results of the detailed balance voltage loss analysis following Rau *et al.*[42,43] as described in the Experimental Methods section. The slightly negative radiative voltage loss for Y6/F_4ZnPc is unphysical and attributed to some uncertainty in the voltage loss analysis as discussed, for example, in fig. S5.

	E_{g} (eV)	$V_{\mathrm{oc}}^{\mathrm{SQ}}$ (V)	$V_{\mathrm{oc}}^{\mathrm{sc}}$ (V)	$V_{\mathrm{oc}}^{\mathrm{rad}}$ (V)	V_{oc} (V)	$\Delta V_{\mathrm{oc}}^{\mathrm{SQ}}$ (V)	$\Delta V_{\mathrm{oc}}^{\mathrm{sc}}$ (V)	$\Delta V_{\mathrm{oc}}^{\mathrm{rad}}$ (V)	$\Delta V_{\mathrm{oc}}^{\mathrm{nr}}$ (V)	Q_{LED}
C_{60}/ZnPc	1.64	1.37	1.32	0.93	0.406	0.27	0.046	0.39	0.52	9.4E-10
Y6/ZnPc	1.38	1.13	1.07	1.05	0.639	0.25	0.054	0.03	0.41	9.3E-08
C_{60}/F_4ZnPc	1.64	1.37	1.32	1.16	0.669	0.27	0.046	0.16	0.49	3.1E-09

Y6/F_4ZnPc	1.38	1.13	1.05	1.07	0.786	0.25	0.073	-0.01	0.28	1.4E-05

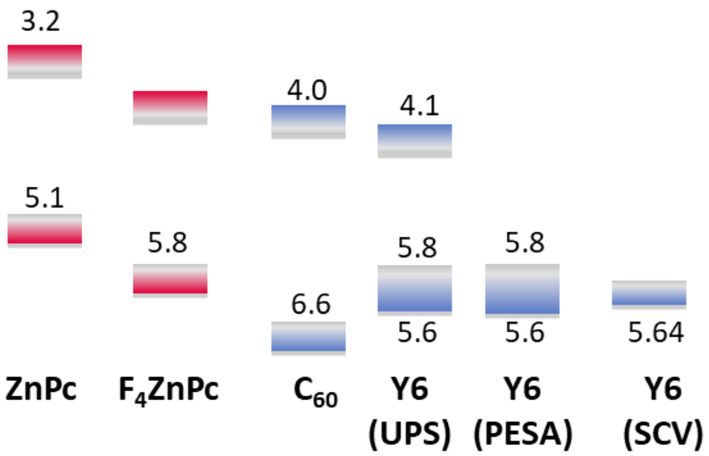

Figure S6: Energy levels taken from literature. Unless noted otherwise, the cited ionization energies[44–46] (IEs) were measured with ultraviolet photoelectron spectroscopy (UPS) and electron affinities[45] (EAs) were measured with low energy inverse photoelectron spectroscopy (LEIPS). Values are determined from the peak onsets and should be taken with caution since they refer to different setups and samples. For Y6, we display a range of IE values reported in literature, which can be partially attributed to different processing conditions (see below). We included values measured via UPS[45,47,48], photoelectron spectroscopy in air (PESA)[45,48,49], and spectroscopic cyclic voltammetry under illumination (SCV)[47]. Conceptually, assigning one value to a material's IE or EA is problematic because a different orientation of a molecule yields different IE values[50], making the measured molecular energy levels a function of the film processing conditions[46], which has been shown for Y6 recently[49].

Even if energy levels are measured carefully on a single-component film that matches the processing conditions of corresponding devices, the values do not necessarily correspond to the energy levels at the donor-acceptor interface in the device[47] because the electrostatic environment is different and dipole[51] and quadrupole fields[45,52] of the other molecular species will cause shifts compared to the experimentally determined value at the interface with vacuum or air. The systematic studies cited here demonstrate an improving understanding of the relation between molecular energy levels measured in neat films and their relation to device parameters. This is supported by promising improvements of experimental probes, such as spectroelechtrochemistry[47], that directly investigate the interfacial molecular energy levels in the device-relevant configuration.

While the consensus on the best way of determining device-relevant molecular energy levels is still evolving, we focus on energy levels directly probed in the device, namely CT state energies and only add some brief notes on molecular energy levels on a qualitative level: Compared to ZnPc, the ionization energy IE of the fluorinated F_4ZnPc donor will be closer to the IE of Y6. If the offset ΔIE at the donor/acceptor interface becomes too small, exciton separation and photocurrent generation become less efficient[45,48,52], which could explain the lower photocurrent of the F_4ZnPc devices. Since the optical gap of F_xZnPc is larger than Y6, the electron affinity offset ΔEA is larger than the ΔIE and we may expect the ΔIE to be limiting for the exciton separation process. Interestingly, for many small molecule pairing, including Y6 and the donors used herein, both species have a quadrupolar moment. This is different to blends of SM:C60 or polymer:SM, where only one species has a non-negligible quadrupole moment, because C60 and polymers both have weak quadrupoles. We thus expect band

bending in the donor and acceptor pairings, whose strength and (energetic) direction depends on the quadrupole sign and strength of both species, adding additional complexity to the diagrams presented in refs. [49,53].

Bibliography

(1) Shaheen, S. E.; Brabec, C. J.; Sariciftci, N. S.; Padinger, F.; Fromherz, T.; Hummelen, J. C. 2.5% Efficient Organic Plastic Solar Cells. *Appl Phys Lett* **2001**, *78* (6), 841–843. https://doi.org/10.1063/1.1345834.

(2) Li, G.; Shrotriya, V.; Huang, J.; Yao, Y.; Moriarty, T.; Emery, K.; Yang, Y. High-Efficiency Solution Processable Polymer Photovoltaic Cells by Self-Organization of Polymer Blends. *Nat Mater* **2005**, *4* (11), 864–868. https://doi.org/10.1038/nmat1500.

(3) Peet, J.; Kim, J. Y.; Coates, N. E.; Ma, W. L.; Moses, D.; Heeger, A. J.; Bazan, G. C. Efficiency Enhancement in Low-Bandgap Polymer Solar Cells by Processing with Alkane Dithiols. *Nat Mater* **2007**, *6* (7), 497–500. https://doi.org/10.1038/nmat1928.

(4) Park, S. H.; Roy, A.; Beaupré, S.; Cho, S.; Coates, N.; Moon, J. S.; Moses, D.; Leclerc, M.; Lee, K.; Heeger, A. J. Bulk Heterojunction Solar Cells with Internal Quantum Efficiency Approaching 100%. *Nat Photonics* **2009**, *3* (5), 297–303. https://doi.org/10.1038/nphoton.2009.69.

(5) He, Z.; Zhong, C.; Su, S.; Xu, M.; Wu, H.; Cao, Y. Enhanced Power-Conversion Efficiency in Polymer Solar Cells Using an Inverted Device Structure. *Nat Photonics* **2012**, *6* (9), 591–595. https://doi.org/10.1038/nphoton.2012.190.

(6) Nguyen, T. L.; Choi, H.; Ko, S. J.; Uddin, M. A.; Walker, B.; Yum, S.; Jeong, J. E.; Yun, M. H.; Shin, T. J.; Hwang, S.; Kim, J. Y.; Woo, H. Y. Semi-Crystalline Photovoltaic Polymers with Efficiency Exceeding 9% in a ∼300 Nm Thick Conventional Single-Cell Device. *Energy Environ Sci* **2014**, *7* (9), 3040–3051. https://doi.org/10.1039/c4ee01529k.

(7) Kawashima, K.; Tamai, Y.; Ohkita, H.; Osaka, I.; Takimiya, K. High-Efficiency Polymer Solar Cells with Small Photon Energy Loss. *Nat Commun* **2015**, *6*. https://doi.org/10.1038/ncomms10085.

(8) Vohra, V.; Kawashima, K.; Kakara, T.; Koganezawa, T.; Osaka, I.; Takimiya, K.; Murata, H. Efficient Inverted Polymer Solar Cells Employing Favourable Molecular Orientation. *Nat Photonics* **2015**, *9* (6), 403–408. https://doi.org/10.1038/nphoton.2015.84.

(9) Zhao, J.; Li, Y.; Yang, G.; Jiang, K.; Lin, H.; Ade, H.; Ma, W.; Yan, H. Efficient Organic Solar Cells Processed from Hydrocarbon Solvents. *Nat Energy* **2016**, *1* (2). https://doi.org/10.1038/NENERGY.2015.27.

(10) Zhao, W.; Qian, D.; Zhang, S.; Li, S.; Inganäs, O.; Gao, F.; Hou, J. Fullerene-Free Polymer Solar Cells with over 11% Efficiency and Excellent Thermal Stability. *Advanced Materials* **2016**, *28* (23), 4734–4739. https://doi.org/10.1002/adma.201600281.

(11) Zhao, W.; Li, S.; Zhang, S.; Liu, X.; Hou, J. Ternary Polymer Solar Cells Based on Two Acceptors and One Donor for Achieving 12.2% Efficiency. *Advanced Materials* **2017**, *29* (2). https://doi.org/10.1002/adma.201604059.

(12) Baran, D.; Ashraf, R. S.; Hanifi, D. A.; Abdelsamie, M.; Gasparini, N.; Röhr, J. A.; Holliday, S.; Wadsworth, A.; Lockett, S.; Neophytou, M.; Emmott, C. J. M.; Nelson, J.; Brabec, C. J.; Amassian, A.; Salleo, A.; Kirchartz, T.; Durrant, J. R.; McCulloch, I. Reducing the Efficiencystabilitycost Gap of Organic Photovoltaics with Highly Efficient and Stable Small Molecule Acceptor Ternary Solar Cells. *Nat. Mater.* **2016**, *16* (3), 363–369. https://doi.org/10.1038/nmat4797.

(13) Zhang, G.; Zhang, K.; Yin, Q.; Jiang, X. F.; Wang, Z.; Xin, J.; Ma, W.; Yan, H.; Huang, F.; Cao, Y. High-Performance Ternary Organic Solar Cell Enabled by a Thick Active Layer Containing a Liquid Crystalline Small Molecule Donor. *J Am Chem Soc* **2017**, *139* (6), 2387–2395. https://doi.org/10.1021/jacs.6b11991.

(14) Zhao, W.; Li, S.; Yao, H.; Zhang, S.; Zhang, Y.; Yang, B.; Hou, J. Molecular Optimization Enables over 13% Efficiency in Organic Solar Cells. *J Am Chem Soc* **2017**, *139* (21), 7148–7151. https://doi.org/10.1021/jacs.7b02677.

(15) Xiao, Z.; Jia, X.; Ding, L. Ternary Organic Solar Cells Offer 14% Power Conversion Efficiency. *Sci Bull (Beijing)* **2017**, *62* (23), 1562–1564. https://doi.org/10.1016/j.scib.2017.11.003.

(16) Yuan, J.; Zhang, Y.; Zhou, L.; Zhang, G.; Yip, H. L.; Lau, T. K.; Lu, X.; Zhu, C.; Peng, H.; Johnson, P. A.; Leclerc, M.; Cao, Y.; Ulanski, J.; Li, Y.; Zou, Y. Single-Junction Organic Solar Cell with over 15% Efficiency Using Fused-Ring Acceptor with Electron-Deficient Core. *Joule* **2019**, *3* (4), 1140–1151. https://doi.org/10.1016/j.joule.2019.01.004.

(17) Cui, Y.; Yao, H.; Zhang, J.; Zhang, T.; Wang, Y.; Hong, L.; Xian, K.; Xu, B.; Zhang, S.; Peng, J.; Wei, Z.; Gao, F.; Hou, J. Over 16% Efficiency Organic Photovoltaic Cells Enabled by a Chlorinated Acceptor with Increased Open-Circuit Voltages. *Nat Commun* **2019**, *10* (1). https://doi.org/10.1038/s41467-019-10351-5.

(18) Liu, Q.; Jiang, Y.; Jin, K.; Qin, J.; Xu, J.; Li, W.; Xiong, J.; Liu, J.; Xiao, Z.; Sun, K.; Yang, S.; Zhang, X.; Ding, L. 18% Efficiency Organic Solar Cells. *Sci Bull (Beijing)* **2020**, *65* (4), 272–275. https://doi.org/10.1016/j.scib.2020.01.001.

(19) Bi, P.; Zhang, S.; Chen, Z.; Xu, Y.; Cui, Y.; Zhang, T.; Ren, J.; Qin, J.; Hong, L.; Hao, X.; Hou, J. Reduced Non-Radiative Charge Recombination Enables Organic Photovoltaic Cell Approaching 19% Efficiency. *Joule* **2021**, *5* (9), 2408–2419. https://doi.org/10.1016/j.joule.2021.06.020.

(20) Cui, Y.; Xu, Y.; Yao, H.; Bi, P.; Hong, L.; Zhang, J.; Zu, Y.; Zhang, T.; Qin, J.; Ren, J.; Chen, Z.; He, C.; Hao, X.; Wei, Z.; Hou, J. Single-Junction Organic Photovoltaic Cell with 19% Efficiency. *Advanced Materials* **2021**, 2102420. https://doi.org/10.1002/adma.202102420.

(21) Zhu, L.; Zhang, M.; Xu, J.; Li, C.; Yan, J.; Zhou, G.; Zhong, W.; Hao, T.; Song, J.; Xue, X.; Zhou, Z.; Zeng, R.; Zhu, H.; Chen, C. C.; MacKenzie, R. C. I.; Zou, Y.; Nelson, J.; Zhang, Y.; Sun, Y.; Liu, F. Single-Junction Organic Solar Cells with over 19% Efficiency Enabled by a Refined Double-Fibril Network Morphology. *Nat Mater* **2022**, *21* (6), 656–663. https://doi.org/10.1038/s41563-022-01244-y.

(22) Zhan, L.; Li, S.; Li, Y.; Sun, R.; Min, J.; Chen, Y.; Fang, J.; Ma, C. Q.; Zhou, G.; Zhu, H.; Zuo, L.; Qiu, H.; Yin, S.; Chen, H. Manipulating Charge Transfer and Transport via Intermediary Electron Acceptor Channels Enables 19.3% Efficiency Organic Photovoltaics. *Adv Energy Mater* **2022**, *12* (39). https://doi.org/10.1002/aenm.202201076.

(23) Xue, J.; Rand, B. P.; Uchida, S.; Forrest, S. R. A Hybrid Planar-Mixed Molecular Heterojunction Photovoltaic Cell. *Advanced Materials* **2005**, *17* (1), 66–71. https://doi.org/10.1002/adma.200400617.

(24) Taima, T.; Toyoshima, S.; Kara, K.; Saito, K.; Yase, K. Control of Measurement Environments for High-Efficiency Organic Photovoltaic Cells. *Japanese Journal of Applied Physics, Part 2: Letters* **2006**, *45* (8–11). https://doi.org/10.1143/JJAP.45.L217.

(25) Sakai, K.; Hiramoto, M. Efficient Organic P-i-n Solar Cells Having Very Thick Codeposited i-Layer Consisting of Highly Purified Organic Semiconductors. In *Molecular Crystals and Liquid Crystals*; 2008; Vol. 491, pp 284–289. https://doi.org/10.1080/15421400802330960.

(26) Wynands, D.; Levichkova, M.; Leo, K.; Uhrich, C.; Schwartz, G.; Hildebrandt, D.; Pfeiffer, M.; Riede, M. Increase in Internal Quantum Efficiency in Small Molecular Oligothiophene: C60 Mixed Heterojunction Solar Cells by Substrate Heating. *Appl Phys Lett* **2010**, *97* (7). https://doi.org/10.1063/1.3475766.

(27) Steinmann, V.; Kronenberg, N. M.; Lenze, M. R.; Graf, S. M.; Hertel, D.; Meerholz, K.; Bürckstümmer, H.; Tulyakova, E. v.; Würthner, F. Simple, Highly Efficient Vacuum-Processed Bulk Heterojunction Solar Cells Based on Merocyanine Dyes. *Adv Energy Mater* **2011**, *1* (5), 888–893. https://doi.org/10.1002/aenm.201100283.

(28) Meiss, J.; Merten, A.; Hein, M.; Schuenemann, C.; Schäfer, S.; Tietze, M.; Uhrich, C.; Pfeiffer, M.; Leo, K.; Riede, M. Fluorinated Zinc Phthalocyanine as Donor for Efficient Vacuum-Deposited Organic Solar Cells. *Adv Funct Mater* **2012**, *22* (2), 405–414. https://doi.org/10.1002/adfm.201101799.

(29) Pandey, R.; Zou, Y.; Holmes, R. J. Efficient, Bulk Heterojunction Organic Photovoltaic Cells Based on Boron Subphthalocyanine Chloride-C 70. *Appl Phys Lett* **2012**, *101* (3), 033308. https://doi.org/10.1063/1.4737902.

(30) Xiao, X.; Bergemann, K. J.; Zimmerman, J. D.; Lee, K.; Forrest, S. R. Small-Molecule Planar-Mixed Heterojunction Photovoltaic Cells with Fullerene-Based Electron Filtering Buffers. *Adv Energy Mater* **2014**, *4* (7). https://doi.org/10.1002/aenm.201301557.

(31) Meerheim, R.; Körner, C.; Leo, K. Highly Efficient Organic Multi-Junction Solar Cells with a Thiophene Based Donor Material. *Appl Phys Lett* **2014**, *105* (6). https://doi.org/10.1063/1.4893012.

(32) Griffith, O. L.; Liu, X.; Amonoo, J. A.; Djurovich, P. I.; Thompson, M. E.; Green, P. F.; Forrest, S. R. Charge Transport and Exciton Dissociation in Organic Solar Cells Consisting of Dipolar Donors Mixed with C70. *Phys Rev B Condens Matter Mater Phys* **2015**, *92* (8). https://doi.org/10.1103/PhysRevB.92.085404.

(33) Che, X.; Chung, C. L.; Hsu, C. C.; Liu, F.; Wong, K. T.; Forrest, S. R. Donor–Acceptor–Acceptor's Molecules for Vacuum-Deposited Organic Photovoltaics with Efficiency Exceeding 9%. *Adv Energy Mater* **2018**, *8* (19). https://doi.org/10.1002/aenm.201703603.

(34) Li, T. Y.; Benduhn, J.; Li, Y.; Jaiser, F.; Spoltore, D.; Zeika, O.; Ma, Z.; Neher, D.; Vandewal, K.; Leo, K. Boron Dipyrromethene (BODIPY) with: Meso -Perfluorinated Alkyl Substituents as near Infrared Donors in Organic Solar Cells. *J Mater Chem A Mater* **2018**, *6* (38), 18583–18591. https://doi.org/10.1039/c8ta06261g.

(35) Wang, C. K.; Che, X.; Lo, Y. C.; Li, Y. Z.; Wang, Y. H.; Forrest, S. R.; Liu, S. W.; Wong, K. T. New D-A-A'-Configured Small Molecule Donors Employing Conjugation to Red-Shift the Absorption for Photovoltaics. *Chem Asian J* **2020**, *15* (16), 2520–2531. https://doi.org/10.1002/asia.202000635.

(36) Ullbrich, S.; Benduhn, J.; Jia, X.; Nikolis, V. C.; Tvingstedt, K.; Piersimoni, F.; Roland, S.; Liu, Y.; Wu, J.; Fischer, A.; Neher, D.; Reineke, S.; Spoltore, D.; Vandewal, K. Emissive and Charge-Generating Donor–Acceptor Interfaces for Organic Optoelectronics with Low Voltage Losses. *Nature Materials*. Nature Publishing Group May 1, 2019, pp 459–464. https://doi.org/10.1038/s41563-019-0324-5.

(37) Yue, Q.; Liu, S.; Xu, S.; Liu, G.; Jiang, Y.; Wang, Y.; Zhu, X. Vacuum-Deposited Organic Solar Cells Utilizing a Low-Bandgap Non-Fullerene Acceptor. *J Mater Chem C Mater* **2022**, *10* (7), 2569–2574. https://doi.org/10.1039/d1tc03954g.

(38) Nikolis, V. C.; Dong, Y.; Kublitski, J.; Benduhn, J.; Zheng, X.; Huang, C.; Yüzer, A. C.; Ince, M.; Spoltore, D.; Durrant, J. R.; Bakulin, A. A.; Vandewal, K. Field Effect versus Driving Force: Charge Generation in Small-Molecule Organic Solar Cells. *Adv Energy Mater* **2020**, *10* (47), 2002124. https://doi.org/10.1002/aenm.202002124.

(39) Nikolis, V. C.; Benduhn, J.; Holzmueller, F.; Piersimoni, F.; Lau, M.; Zeika, O.; Neher, D.; Koerner, C.; Spoltore, D.; Vandewal, K. Reducing Voltage Losses in Cascade Organic Solar Cells While Maintaining High External Quantum Efficiencies. *Adv Energy Mater* **2017**, *7* (21). https://doi.org/10.1002/aenm.201700855.

(40) Cnops, K.; Rand, B. P.; Cheyns, D.; Verreet, B.; Empl, M. A.; Heremans, P. 8.4% Efficient Fullerene-Free Organic Solar Cells Exploiting Long-Range Exciton Energy Transfer. *Nat Commun* **2014**, *5*. https://doi.org/10.1038/ncomms4406.

(41) Vandewal, K.; Benduhn, J.; Nikolis, V. C. How to Determine Optical Gaps and Voltage Losses in Organic Photovoltaic Materials. *Sustainable Energy and Fuels*. Royal Society of Chemistry March 1, 2018, pp 538–544. https://doi.org/10.1039/c7se00601b.

(42) Jungbluth, A.; Kaienburg, P.; Riede, M. Charge Transfer State Characterization and Voltage Losses of Organic Solar Cells. *Journal of Physics: Materials* **2021**. https://doi.org/10.1088/2515-7639/ac44d9.

(43) Rau, U.; Blank, B.; Müller, T. C. M.; Kirchartz, T. Efficiency Potential of Photovoltaic Materials and Devices Unveiled by Detailed-Balance Analysis. *Phys. Rev. Appl* **2017**, *7* (4), 044016. https://doi.org/10.1103/physrevapplied.7.044016.

(44) Schwarze, M.; Tress, W.; Beyer, B.; Gao, F.; Scholz, R.; Poelking, C.; Ortstein, K.; Günther, A. A.; Kasemann, D.; Andrienko, D.; Leo, K. Band Structure Engineering in Organic Semiconductors. *Science (1979)* **2016**, *352* (6292), 1446–1449. https://doi.org/10.1126/science.aaf0590.

(45) Karuthedath, S.; Gorenflot, J.; Firdaus, Y.; Chaturvedi, N.; De Castro, C. S. P.; Harrison, G. T.; Khan, J. I.; Markina, A.; Balawi, A. H.; Peña, T. A. Dela; Liu, W.; Liang, R. Z.; Sharma, A.; Paleti,

S. H. K.; Zhang, W.; Lin, Y.; Alarousu, E.; Anjum, D. H.; Beaujuge, P. M.; De Wolf, S.; McCulloch, I.; Anthopoulos, T. D.; Baran, D.; Andrienko, D.; Laquai, F. Intrinsic Efficiency Limits in Low-Bandgap Non-Fullerene Acceptor Organic Solar Cells. *Nat Mater* **2021**, *20* (3), 378–384. https://doi.org/10.1038/s41563-020-00835-x.

(46) Schwarze, M.; Schellhammer, K. S.; Ortstein, K.; Benduhn, J.; Gaul, C.; Hinderhofer, A.; Perdigón Toro, L.; Scholz, R.; Kublitski, J.; Roland, S.; Lau, M.; Poelking, C.; Andrienko, D.; Cuniberti, G.; Schreiber, F.; Neher, D.; Vandewal, K.; Ortmann, F.; Leo, K. Impact of Molecular Quadrupole Moments on the Energy Levels at Organic Heterojunctions. *Nat Commun* **2019**, *10* (1), 2466. https://doi.org/10.1038/s41467-019-10435-2.

(47) Neusser, D.; Sun, B.; Tan, W. L.; Thomsen, L.; Schultz, T.; Perdigón-Toro, L.; Koch, N.; Shoaee, S.; McNeill, C. R.; Neher, D.; Ludwigs, S. Spectroelectrochemically Determined Energy Levels of PM6:Y6 Blends and Their Relevance to Solar Cell Performance. *J Mater Chem C Mater* **2022**, *10* (32), 11565–11578. https://doi.org/10.1039/D2TC01918C.

(48) Bertrandie, J.; Han, J.; De Castro, C. S. P.; Yengel, E.; Gorenflot, J.; Anthopoulos, T.; Laquai, F.; Sharma, A.; Baran, D. The Energy Level Conundrum of Organic Semiconductors in Solar Cells. *Advanced Materials* **2022**, *34* (35). https://doi.org/10.1002/adma.202202575.

(49) Fu, Y.; Lee, T. H.; Chin, Y. C.; Pacalaj, R. A.; Labanti, C.; Park, S. Y.; Dong, Y.; Cho, H. W.; Kim, J. Y.; Minami, D.; Durrant, J. R.; Kim, J. S. Molecular Orientation-Dependent Energetic Shifts in Solution-Processed Non-Fullerene Acceptors and Their Impact on Organic Photovoltaic Performance. *Nat Commun* **2023**, *14* (1), 1870. https://doi.org/10.1038/s41467-023-37234-0.

(50) Duhm, S.; Heimel, G.; Salzmann, I.; Glowatzki, H.; Johnson, R. L.; Vollmer, A.; Rabe, J. P.; Koch, N. Orientation-Dependent Ionization Energies and Interface Dipoles in Ordered Molecular Assemblies. *Nat Mater* **2008**, *7* (4), 326–332. https://doi.org/10.1038/nmat2119.

(51) Li, X.; Zhang, Q.; Yu, J.; Xu, Y.; Zhang, R.; Wang, C.; Zhang, H.; Fabiano, S.; Liu, X.; Hou, J.; Gao, F.; Fahlman, M. Mapping the Energy Level Alignment at Donor/Acceptor Interfaces in Non-Fullerene Organic Solar Cells. *Nat Commun* **2022**, *13* (1). https://doi.org/10.1038/s41467-022-29702-w.

(52) Markina, A.; Lin, K.; Liu, W.; Poelking, C.; Firdaus, Y.; Villalva, D. R.; Khan, J. I.; Paleti, S. H. K.; Harrison, G. T.; Gorenflot, J.; Zhang, W.; De Wolf, S.; McCulloch, I.; Anthopoulos, T. D.; Baran, D.; Laquai, F.; Andrienko, D. Chemical Design Rules for Non-Fullerene Acceptors in Organic Solar Cells. *Adv Energy Mater* **2021**, *11* (44), 2102363. https://doi.org/10.1002/aenm.202102363.

(53) Jungbluth, A.; Kaienburg, P.; Lauritzen, A. E.; Derrien, T.; Riede, M. Probing the Energy Levels of Organic Bulk Heterojunctions by Varying the Donor Content. *APL Mater* **2023**, *11* (6). https://doi.org/10.1063/5.0148191.
